# Effect of using deflector in the distributor head of a pneumatic seed drill on the oat seed sowing unevenness

**DOI:** 10.1038/s41598-023-42476-5

**Published:** 2023-09-19

**Authors:** Łukasz Gierz, Piotr Markowski, Dariusz Jan Choszcz, Dawid Wojcieszak

**Affiliations:** 1 Institute of Machine Design, Faculty of Mechanical Engineering, Poznań University of Technology, Ul. Piotrowo 3, 60-965 Poznan, Poland; 2 Department of Heavy Duty Machines and Research Methodology, University of Warmia and Mazury, Oczapowskiego 11, 10-719 Olsztyn, Poland; 3 Department of Biosystems Engineering, Poznan University of Life Sciences, Wojska Polskiego 50, 60-627 Poznan, Poland

**Keywords:** Mechanical engineering, Engineering, Civil engineering

## Abstract

This paper presents an innovative solution for a distributor head equipped with a deflector (controlled plate)—intended to change the tilt angle (realignment) of the pneumatic seed drill distributor head cover. We compared two qualitative parameters of seed sowing, coefficient of variation and coefficient of lateral unevenness of seed sowing (*δ*). Values were obtained on the test stand with an innovative deflector built into the distributor head at three angles of inclination (0°, 5° and 10°). Statistical analyses revealed a significant effect of airflow velocity and deflector angle, which corrects the deviation from the vertical plane of the distributor head, on the uniformity of seed sowing. In addition, regression equations were determined to predict the quality of the seed sowing process. The developed and manufactured innovative distributor head with a deflector that tilts in two planes, designed to improve the distribution evenness of the air stream transporting seed to individual coulters in pneumatic seed drills, received a positive review. The use of a deflector with automatic control of its position angle, correcting the deviation of the distributor head from a vertical plane in pneumatic seed drills improves the uniformity of seeding. Therefore, it is reasonable to use this solution for new pneumatic seed drills and those in use on soils with different relief (undulating surface). Moreover, the solution fits in with modern agricultural manufacturing in accordance with the ideas of precision agriculture.

## Introduction

Pneumatic seed drills are equipped with a primary air system that transports the seed from the main seed tank to the seed conduits that end in coulters. Distribution of the main seed stream to the individual seed conduits takes place in a separation device, called a distributor head. The head should be designed to divide the main seed stream into equal unit streams, which travel to the rows in the soil through seed tubes and coulters. The distribution principle of the heads is based on the vortex interaction of the turbulent air stream with the seed and the resulting stochastic distribution of the seed^1^.

Pneumatic seed drills, despite their numerous advantages including high efficiency, maneuverability, easy and fast loading of seed^2–4^, have disadvantages. The most important of them is low lateral sowing evenness^5,6^, especially in terrain with varied terrain (hills)^1^.

In the literature there are many papers concerning the causes of unevenness of seed sowing performed with pneumatic seed drills^7,8^. There are also studies which prove that uniform distribution of seeds in the soil has a positive effect on the crop quantity and quality by improving utilization of sun rays and water by growing plants^9^. Moreover, intraspecific competition is reduced as plants have a similar life space. Authors of these studies use various indicators, test procedures^10,11^ and methods of seed uniformity assessment^10–14^ to evaluate the process of machine seeding. Pippig has analyzed the influence of technical parameters of pneumatic seeding system units and found that the diffuser tube length should be about 350 mm to maintain acceptable uniformity of seeding^15^. Subsequent studies indicate the distribution of air flow velocities at the outlet of the seed (pneumatic) tube, which is necessary to validate the mathematical model of two-phase flow mechanics in pneumatic seed drill systems^5^, and proposes minimum seed transport velocities^8,16^. From the analysis of the literature, it appears that the uniformity of seed distribution is also influenced, though minimally (2°–3°), by inclination of the seed-air stream distributing head^17^. There is also research whose results are mathematical models describing evenness of the seed distribution in the distributor head^5,18^. Other researchers work on improving or developing new sowing system designs^5^ as well as using electronic optical systems to control the sowing process^19^. Xiaolong et al.^20^ designed and tested a new pneumatic precision centralized seed dispenser with internal seed loading, which sows six rows simultaneously. The authors explored the influence of design and operational parameters on sowing evenness. Karayel et al.^21^ developed a new helicoidal seed tube where falling seeds move along a seed spiral which improves seed distribution uniformity. The tests were conducted for two species of wheat and barley grains. The values of variability coefficient for the grain species changed from the level of 118.4% and 139.5% to the level of 77.2% and 70.6%^21^, respectively. In another study the authors developed and tested in field conditions a contactless, self-priming system for wheat seed injection. The value of seed distribution uniformity variability coefficient was 11.3%^22^. Xi et al^23^ discussed a different approach to the issue of seed distribution uniformity improvement by proposing a seed sowing system without seed delivery tubes whose clearance is 5.0–8.8 cm. The best qualitative parameters of seed sowing were found for 7.0 cm clearance tubes—sowing accuracy was 83.84%, whereas the seed distribution uniformity variability coefficient was 14.68%. The research, however, did not consider a varied terrain (slope). Some simulation research on the seed mixture movement and the air stream coming out of the dispenser was conducted by Zhou et al.^24^, Xiaolong et al.^19,25^ and Bourges et al.^26^. That research involved distribution of the seed-air stream flow in the distributor head. Unfortunately, the authors did not investigate evenness of the dosed air stream distribution with the head tilted from the vertical—head tilt. Kumar et al. carried out research on the effect of the distributor head shape, outlet air stream velocity and sowing unit operation speed on seed distribution evenness. The authors indicated that sowing evenness is influenced by the velocity of the air stream transporting the seed, sowing rate, and the seed physical properties. From the literature analysis, it can be concluded that the research results and their scope are not conclusive and do not exhaust the subject of seed sowing evenness in machine sowing. Therefore, it may be stated that there is still a problem of even seed-air stream flow distribution, especially when sowing seeds in soils with varying terrain—especially on slopes above 10°. According to the research conducted so far^27,28^, the deviation from the vertical of a mechanical-pneumatic seed drill distributor head has a significant effect on the seed flow distribution uniformity inside its head, and the distributor head deviation from the vertical in the range of 0°–20° significantly worsens the coefficient of lateral seed sowing unevenness, up to 14%^27^. Gierz Łukasz and Markowski Piotr^29^ achieved similar correlations for sowing oat seeds. The distributor head deviation from the vertical by an angle of 10° resulted in a 31% evenness deterioration of oat seed sowing (evenness of seed and air stream distribution in the distributor head) from a value of 10.36% at the vertical position of the distributor head to a value of 13.55% at the deviation by an angle of 10°. Yatskul et al.^30^ noticed a similar relationship when sowing wheat seed and tilting the distribution head by an angle from 0° to 22°.

For the selected orientation, the head cover tilt in relation to the diffusion tube vertical axis, can result in more seed being carried in that direction, i.e. asymmetrical seed-air stream distribution. Distributor heads of commonly used pneumatic drills usually are not equipped with seed direction control, and it is not possible to change the seed reflection angle. Another disadvantage of assessing the machine sowing quality noted by the authors is the use of several methods (field and laboratory) to estimate the seed sowing evenness, which makes it difficult and sometimes even impossible to compare the results presented in the respective articles. Therefore, it is not always possible to make an unambiguous evaluation of the influence of the sowing unit individual design and operational factors on the seed sowing evenness. It needs, however, to be noted that regulations included in ISO (7256-1:1984) 28 norm are helpful for unification of methods used for evaluation of seed sowing quality and make it possible to compare the results of variability coefficient (CV) for different grain species^31^.

The authors present evaluation of lateral seed distribution evenness with a pneumatic seed drill using an innovative method of correcting the setting of the distributor head cover of the pneumatic sowing system by means of a controlled plate (deflector), which makes it possible to control the distribution of the seed-air stream by changing the seed reflection angle. To be able to compare the obtained results with those presented in the literature, two coefficients were used to assess the quality of seed sowing—coefficient of variation CV and coefficient of lateral seed sowing unevenness *δ.*

## Material and study methods

### Head with deflector

**Figure 1:**
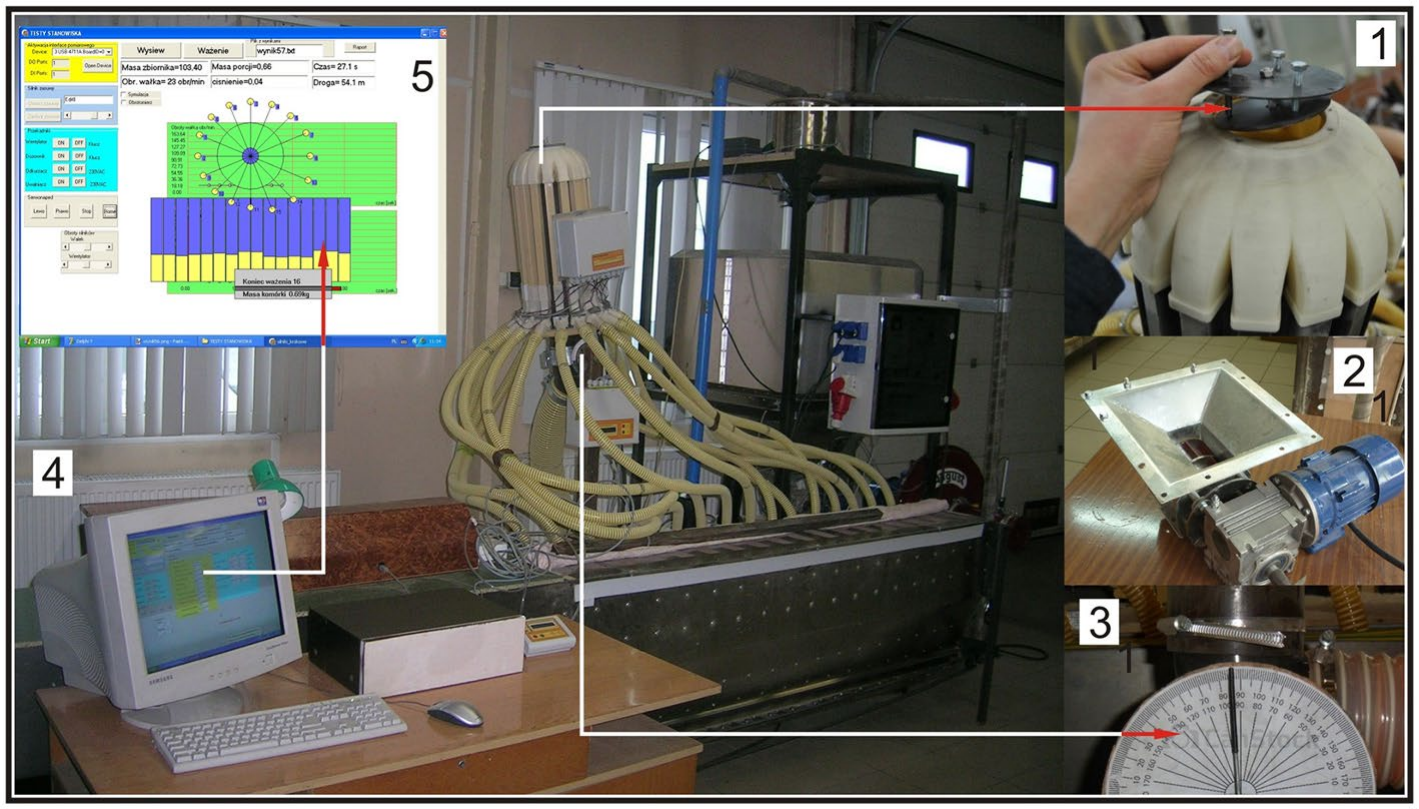
View of the research stand: 1—pneumatic seed distributor head with innovative deflector that tilts in two planes, 2—seed dispensing unit, 3—protractor for measuring the angle of deviation from the vertical of the distribution head, 4—computer with control and steering software, 5—a dialogue form of the program controlling the measuring facility.

The subject of the research is an innovative seed-air stream distributor head with an adjustable angle deflector, made according to patent claim no. PL.230492^28^. The deflector's design allows it to be tilted in two planes—in two perpendicular axes. The deflector tilt angle from the distributor head parallel plane is controlled by three screws numbered 2, 3, 4 (Fig. 1). In practice, this solution enables the deflector to be kept parallel to the plane of the distributor head.

**Figure 2:**
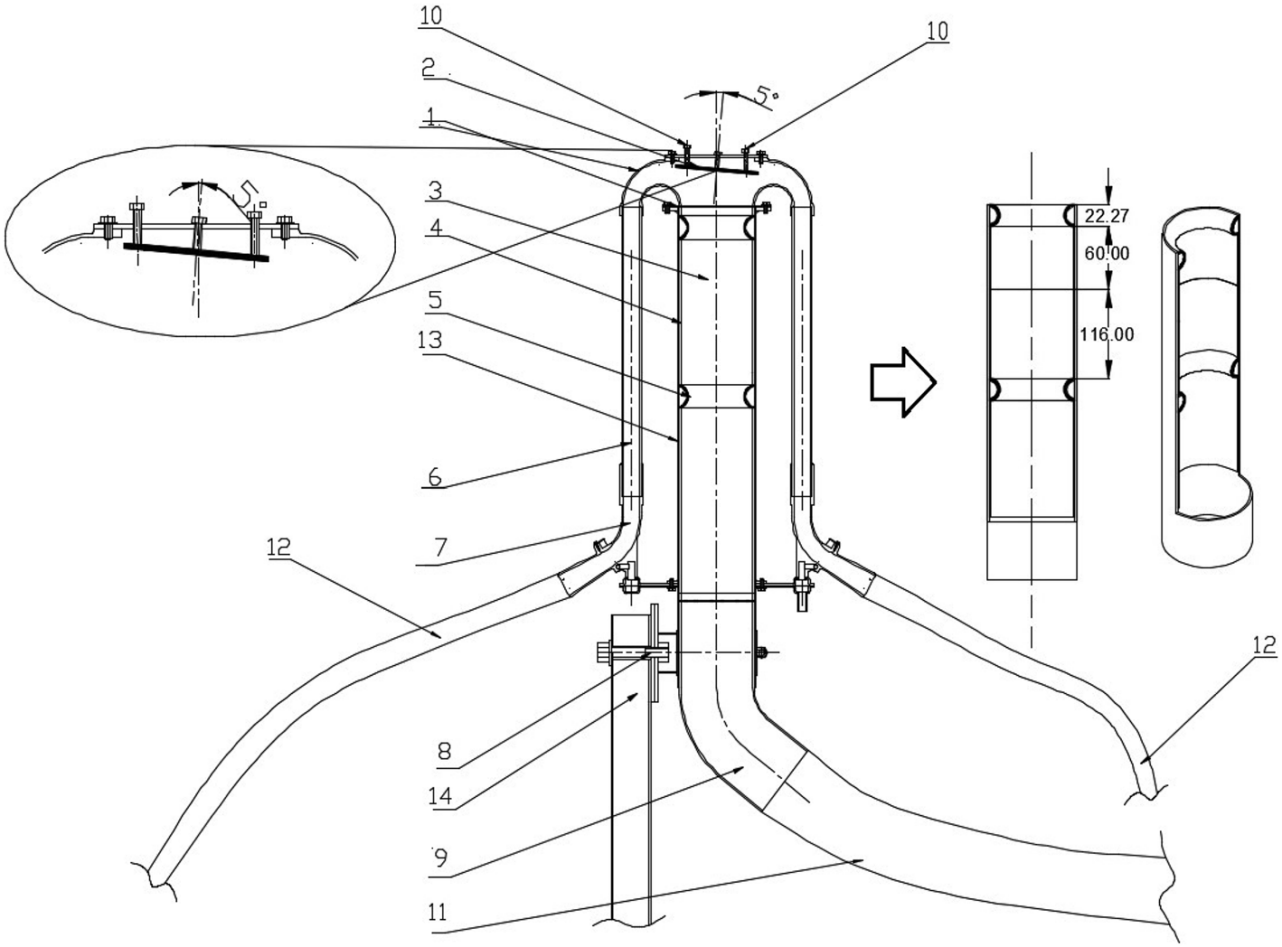
Schematic of the distribution head with the novel deflector including the location of the diffuser rings and spacer sleeves in the diffuser: 1—stream divider, 2—tilt deflector, 3—diffuser, 4—spacer sleeve, 5—diffuser ring, 6—propulsion tube, 7—angle joint with flow sensor, 8—head tilt system, 9—supply angle joint, 9—bracket, 10—adjusting screws, 11—main supply conduit, 12—sowing conduits, 13—diffuser support tube, 14—distribution head support.

The stream divider and diffuser rings were made using Rapid Prototyping method^32–34^. The distributor head diffuser, which scatters the seed, is made of two diffuser rings and a spacer sleeve (Fig. 2). To carry out the tests, the diffuser was deliberately fitted with two diffuser rings instead of the standard nine diffuser rings arranged along the entire length of the diffuser. This design measure was applied to reduce the seed distribution evenness, i.e. to increase the value of the coefficient of seed distribution unevenness, in order to capture more clearly the effect of the deflector on the seed distribution evenness.

The height of the diffuser rings used in the diffuser was 22.27 mm, between which two spacer sleeves of 60 mm and 116 mm were fitted, allowing the diffuser rings to be distant from each other by 176 mm (Fig. 2).

**Figure 3:**
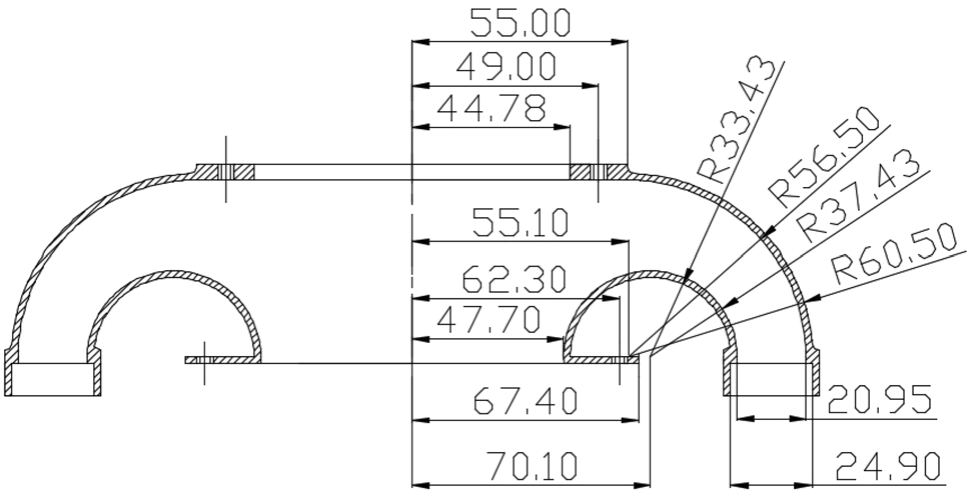
Seed-air stream distributor cross-section with the basic dimensions (mm).

Following the studies of the head tilt effect and ring placement variants on the diffuser^29^ the dimensions of the stream divider were modified to incorporate a novel deflector. The new dimensions of the stream divider are shown in Fig. 3.

**Figure 4:**
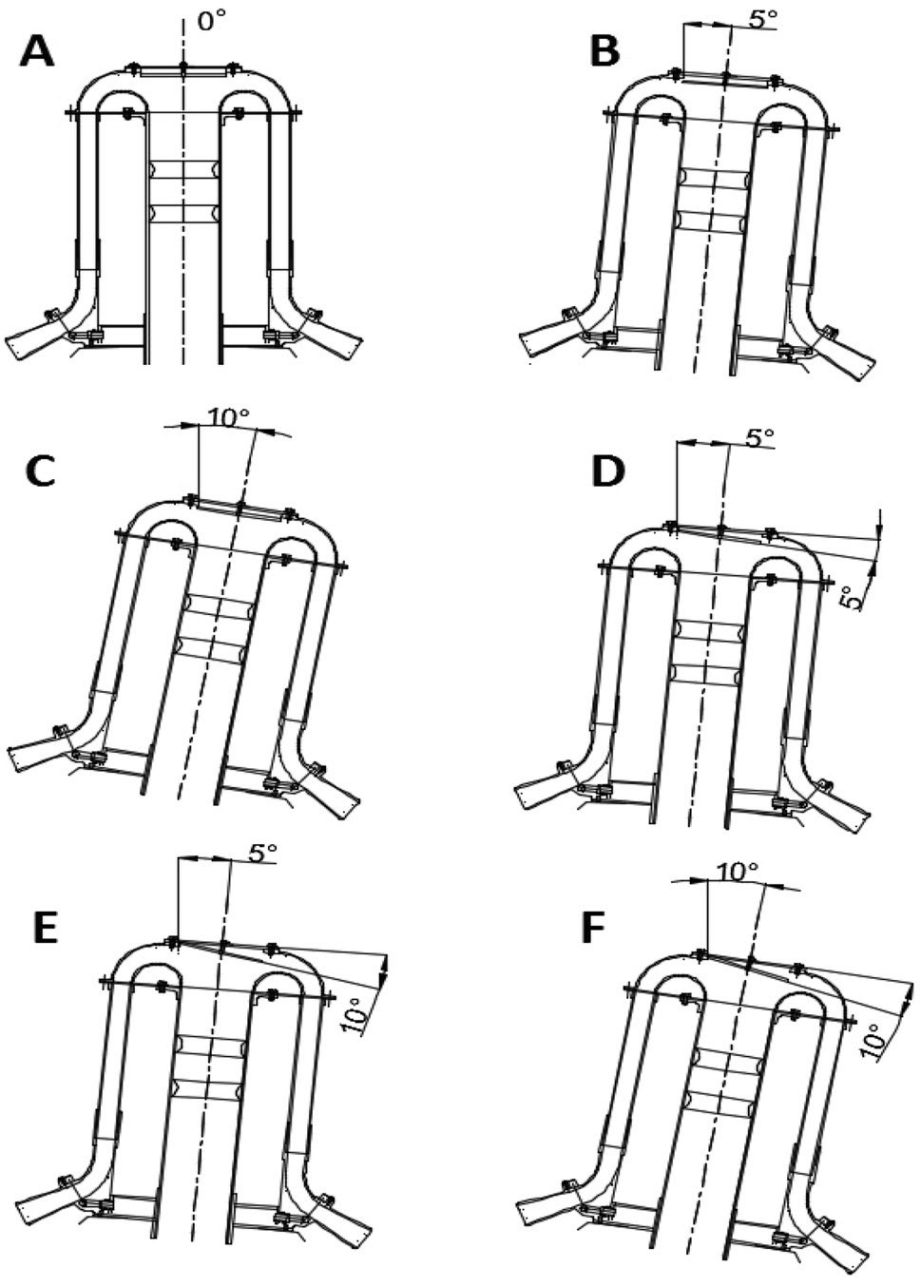
Schematic of the adjusted tilt angles of the distributor head and the deflector.

On a laboratory bench a deflector in the seed distributor head can be adjusted to three positions. The distribution head and deflector angles relative to the distribution head plane are shown in Table 1. For a clear illustration of the distribution head and deflector alignment, it is further illustrated in Fig. 4.

**Table 1:** The deflector and distribution head angles.

Position^a^	Deflector angle *d*_*α*_ (°)	Distribution head angle *h*_*β*_ (°)
A	0	0
B	0	5
C	0	10
D	5	5
E	10	5
F	10	10

^a^Setting option for the head and deflector position.

### Testing rig

**Figure 5:**
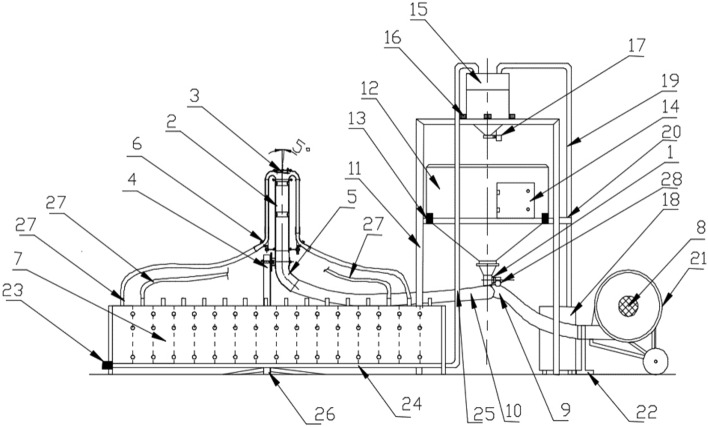
Schematic of a test rig with innovative deflector: 1—dispensing unit with injector, 2—distributor head with tilt deflector, 3—deflector, 4—distributor head tilt system, 5—supply conduit main angle joint, 6—seed flow sensor connector, 7—seed collection box divided into 16 chambers, 8—main fan, 9—air stream supply conduit, 10—the main feed conduit for the seed-air mixture, 11—main chassis, 12—seed box, 13—tensometric seed box weight, 14—steering box, 15—cyclone of the batch metering system, 16—tensometric weight scales for measuring the weight of seed batches, 17—closing flap for the seed cyclone outlet, 18—suction fan, 19—suction (air) conduit, 20—suction conduit bracket, 21—AC electric motor powering the main fan, 22—main fan support, 23—stepper motor for the batch ejection system, 24—collector conduit, 25—suction conduit (seed-air mixture), 26—distribution head support, 27—seed conduits, 28—AC motor with geared motor for dispenser.

Research on the innovative distributor head of a pneumatic seed drill with a deflector tilted in two planes was conducted on a special laboratory bench built at the Department of Mechanical Engineering of the Poznan University of Technology as part of project no. N R03 0021 06/2009^35^ (Fig. 5). The subject of the research is an innovative deflector (2) mounted in the upper cover of the seed-air distributor (1), with tilt angle adjustable (from 0° to 10°) through adjustment screws (10). Stream divider (1) attached to diffuser (3) built of support tube (13) spacer sleeves (4) and diffuser rings (5), is connected to supply angle joint (9) and supply conduit (11). The distributor head with deflector is attached to a distributor head support (14) with tilting system (8) that allows the distributor head with deflector to be tilted from − 20° to + 20° from the vertical. The seed is transported from stream divider (1) with tilted deflector plane (2) through propulsion tube (6) and the connector with flow control sensors to the seed conduits (12).

The laboratory test bench for evaluating the quality of seed sowing (Fig. 4) was constructed on the model of a 16-coulter universal pneumatic row seed drill. It consists of the main chassis (11) on which seed box (12) is suspended on four tensometric weight sensors (13). The accuracy of seed weighing with the tensometric weight scale is ± 0.01 kg. Mounted to the lower wall of the seed box is a dispensing unit with injector (1) driven by a 0.37 kW AC motor with an MR40 geared motor made by Kacperek Polska with ratio i = 30 (28), designed to draw the seed into the main seed-air conduit (10) with internal diameter of 80 mm, which transports it in the air stream through a supply angle joint made of PVC material with 15° tilt angle (5) to the distribution head with tilt deflector (2). The seeds separated and directed in the distributor head with tilt deflector (2) are further transported through seed flow sensor connector (6) and seed conduits (27) with internal diameter of 25 mm to a collection box divided into 16 chambers (7). Each collection box chamber (7) is opened by a cam mechanism driven by BG 65X25 SI servomotor made by Dunkermotoren (23). After opening box chambers (7), the seeds fall down into the collection conduit (26) and are then sucked through under pressured system via the suction conduit (25) into the cyclone of the batch metering system (15). The vacuum is generated by 2G2E0032CM suction fan made by Drainvac, Quebec, Canada (18) connected to cyclone (15) through suction (air) conduit (19) attached by suction conduit bracket (20) to the main chassis (11). Cyclone of the batch metering system (15) is suspended on three tensometric weight sensors of batch metering system (16) with measuring accuracy of ± 0.005 kg and closed at the bottom with a flap (17). The air stream is generated by the main fan (8) with the symbol T 378/1 manufactured by POM Augustów, Poland, which is powered by a 5.5 kW AC electric motor capable of generating an air stream velocity from 5 to 40 m/s, with a capacity of 0.680 m^3^/s and a pressure of 6 kPa^29^ (21) The main fan (8) is connected to the dosing unit with injector (1) via airflow supply conduit (9).

The laboratory test bench is also equipped with a unit for automatic weighing of the quantity of seed sown, a 16-chamber seed collection box and a computer with software for data collection and calculation of the coefficient of lateral seed sowing unevenness.

In this study, oat seed Lion with a thousand-seed weight of 32.2 g ± 0.62 and moisture content of 9.9% was used. The research material came from Main Seed Warehouse Top Farms Seeds, Production Plant in Runów located in Greater Poland Province. Based on the announcement of the Marshal of the Sejm of the Republic of Poland on the announcement of the uniform text of the Act on the legal protection of plant varieties of January 22, 2021 (Journal of Laws of 2021, item 213) and the breeder's declaration that the indicated variety: Lion (oat) is law protected by the breeder, the authors have received this permission. The breeder agrees to obtain the above-mentioned plant material, which complies with the national guidelines from Main Seed Warehouse Top Farms Seeds, Production Plant in Runów located in Greater Poland Province. The authors, with the breeder's consent, may use its plant material only for the purposes of scientific and research activities, including carrying out tests of e.g. sowing simulation on a laboratory stand with an innovative distributor head with a deflector. Seeds were sown at a constant rate of 250 kg/ha.

Once the laboratory experiment (one measuring cycle) is completed on the designed test rig, the 16 chambers of seed collecting box (7) are opened sequentially and their contents are weighed and returned in a closed circuit to seed box (12).

Sliders are installed in the chamber bottoms of collection box (7) and are opened and closed by a cam mechanism driven by toothed belt through servomotor (23). A special program for the laboratory rig written in Embarcadero's RAD Delphi 2010 environment controls the servomotor (23), the main fan supply motor (21), suction fan (18), and dispenser drive motor (28).

Collector pipe is located along the sixteen-chamber box, into which the seeds enter when the sliders are opened. The seeds then are transported by air stream through the suction conduit from the collector pipe to the separating cyclone located above seed box (12). The conical bottom of separation cyclone (15) is closed from below by flap (17). It relies on three tensometric sensors, which measure separately the weight of seeds from each chamber in collection box (7) with an accuracy of ± 0.005 kg. Cyclone flap (17) is closed by the vacuum in the system with a force of approx. 500 N during the seed transport from collection box (7), then after suction blower (18) is switched off, the conical bottom of the cyclone drops onto the strain gauges. Cyclone flap (17) is locked electromagnetically by the control system^29,35^. The whole measurement cycle of the sown seed weight is completely automated and after weighing the seed in all sixteen chambers of the collection box, a test report is generated, and the results are stored in .txt files.

The laboratory test bench with the innovative deflector is equipped with a control system, in which the measurement cycle is programmed according to the following scheme:

1. Read the test parameters written into the control system interface: airflow rate, sowing time;
2. Turn on the main fan motor and wait for its speed to stabilize;
3. Make sure that the cam mechanism is before chamber 1;
4. Regulate the fan shaft speed so that the assumed airflow speed is achieved at the inlet of the seed dosing unit;
5. Start the sowing (dosing) unit and set the desired speed of the sowing (dosing) shaft;
6. Wait 5 s. for the sowing conditions to be established;
7. Direct the seed into the distribution head with the innovative deflector;
8. Keep sowing for a set time—according to the test plan, about 10–600 s.
9. Switch off the sowing unit drive and after 5 s switch on the main fan motor;
10. Start the suction fan;
11. Check the vacuum value in the batch measuring system cyclone;
12. Open another collection box slider;
13. Wait 30 s;
14. Turn off the suction fan;
15. Read the weight of the seed sown from a tensiometer weight scale measuring the weight of seed batch;
16. Read the weight of all the seed from the tensiometer weight of the seed box;
17. Open the flap that closes the seed outlet in the cyclone;
18. If the extreme position of cam mechanism carriage is reached, return to chamber no.1;
19. Compile the measurement results and save them in a database.

### Laboratory bench control system

**Figure 6:**
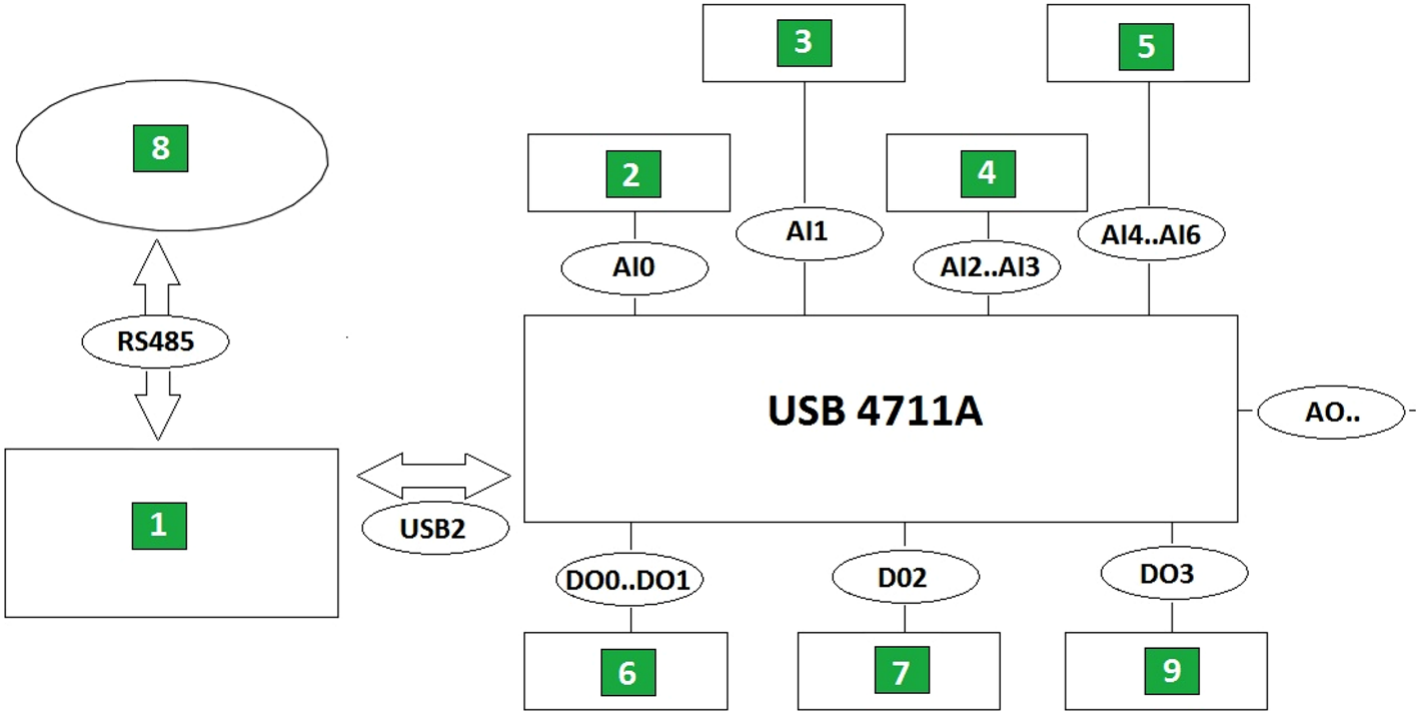
Block diagram of the laboratory bench control system^41^: 1—computer or control unit, 2—unit weighing seed batches, 3—seed box weighing unit, 4—sensor for the tank-opening trolley, 5—air pressure sensor, 6—tank-opening trolley motor, 7—main fan motor, 8—seed-counting unit, 9 —working speed sensor (sowing speed).

The most important component of the laboratory bench for studying the effect of using a deflector in the seed distributor head is a dedicated control system. The base of the control system is a computer with an operating system, equipped with a written program created in RAD Delphi 2010 environment made by Embarcadero company, using measurement libraries provided by Advantech company. The computer program communicates with the environment through USB 4711A measurement interface made by Advantech and through additional coupling systems, including electromagnetic relays and optocouplers^35^. The measuring interface is equipped with a terminal block, where serial transmission conduits (USB2), four digital outputs (DO0…DO3) and six analogue inputs (AI0…AI6) are used to control the device (Fig. 6). A modified position sensor for the tank-opening trolley was used during the tests. The control computer communicates with the seed-counting unit by RS485 serial link during the tests.

The software uses a proprietary graphics and mathematics library, graphics_v3, which complement the control system^29^. The software sets out to divide the entire measurement cycle into two stages: 1—the seed sowing procedure, 2—the procedure of weighing seed sown into individual chambers of the collection box. Both stages are fully automated. Measurement procedure 1 (seed sowing) consists of inputting fixed parameter values in accordance with the test plan after the main dialogue form appears i.e. sowing time. The sowing speed is read from working speed sensor (9) and has also been presumed to be constant. The length of the measuring distance is determined by the sowing time and working speed (sowing rate) specified in the test plan. Measurement procedure 2 (weighing of the individual compartments of the collection tray) involves cyclic opening of the individual box chambers, weight measurement and discharge into the seed box, and the recording of the measured values of the seed weight in the program memory to create a report.

### Calculation of seed sowing quality coefficient values (CV and δ)

**Figure 7:**
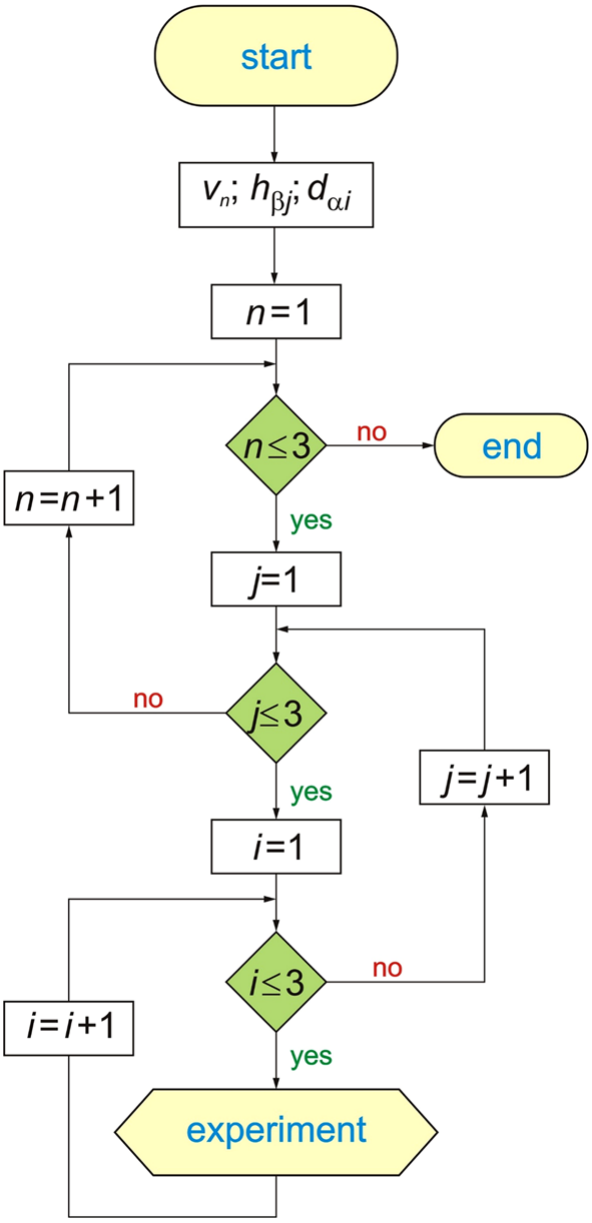
Block diagram of the laboratory bench control system^41^: 1—computer or control unit, 2—unit weighing seed batches, 3—seed box weighing unit, 4—sensor for the tank-opening trolley, 5—air pressure sensor, 6—tank-opening trolley motor, 7—main fan motor, 8—seed-counting unit, 9 —working speed sensor (sowing speed).

There were three repetitions of the experiment for all combinations of variable parameters according to the algorithm shown in Fig. 7.

For the purposes of this study, the following parameters were adopted:

- Constant:
  sowing rate of 250 kg/ha;
  sowing speed 8 km/h;
  sowing time 10 min.
- Variables:
  air velocity in three ranges: 12.22 m/s, 14.44 m/s, 16.66 m/s;
  distribution head tilt: 0°, 5°, 10°;
  deflector tilt: 0°, 5°, 10°.
- Actual parameters:
  coefficient of variation *CV* (%);
  coefficient of lateral seed sowing unevenness *δ* (%).

The quality of sowing (seed distribution evenness) is evaluated based on the values of transverse and lateral unevenness of seed sowing. The indicator of transverse seed sowing *δ*^36^ is frequently determined as variability coefficient (*CV*)^37^, defined as a ratio of square deviation of average distance between seeds sown in particular rows to the average distance determined for the whole seed drill. This indicator can be also determined as a ratio of square deviation of the seed average weight falling on a measurement distance in individual rows to an average weight for the whole seed drill. In turn, an indicator of lateral unevenness of seed sowing is defined as a ratio of square average deviation in a row to average deviation in a row^38^. The indicator of lateral seed sowing unevenness is commonly defined for point seed drills^38^.

To determine the effect of using a deflector in the distributor head on the uniformity of seed-air distribution into individual coulters, coefficient of variation (CV) and coefficient of lateral seed sowing unevenness *δ* were applied. Coefficient of variation (CV) was calculated from relation (1), according to ISO-7256/2^37^:

1 $$ CV = \frac{S}{X} \cdot 100 \left( \% \right); $$

where *S*—standard deviation of the average seed weight from three repetitions for a single coulter, *X*—average weight of seed collected from all coulters.

The coefficient value of lateral seed sowing unevenness *δ* (%) was determined from Eq. (2), according to PN-84/R-55050^36^:

2 $$ \delta = \frac{{\frac{{\sqrt {\sum \left( {\Delta q_{i} } \right)^{2} } }}{i}}}{{q_{sr} }} \cdot 100 \left( \% \right); $$

where *δ*—coefficient of lateral seed sowing unevenness (%). *q*_*sr*_—average weight of oat seed sown in the ith container (kg), *∆q*_*sr*_—the average deviation in weight of seed sown into seed containers (kg).

The air velocity was set in 3 ranges: 12.22 m/s, 14.44 m/s, 16.66 m/s due to the oats seed critical velocity of 8.03 m/s^39^.

### Statistical calculations

Measurement results were statistically analyzed. A correlation analysis was conducted to determine the strength and nature of the relationship between the independent variables and the dependent ones. The following null hypothesis was verified H_0_: the correlation coefficients between the variables are equal to zero. When the values of the correlation coefficients showed a strong relationship between the independent variables and the dependent ones, rectilinear regression equations were determined at a further stage to describe the nature of the relationship. Fifth-degree curvilinear, multiple, multivariate polynomial, second-degree polynomial and power equations were tested as well, followed by regression analysis with a posteriori elimination and stepwise selection procedures to determine the form of the regression equation describing the most accurate fit of the empirical data to the model. Regression function fit assessment was determined, and description quality analyses were performed by removing irrelevant variables from the equations. The calculated value of the F-Snedecor statistic and the probability of exceeding it, the multiple correlation coefficient, the value of the determination coefficient, the standard deviation of the residuals and the coefficient of random variation were used as criteria for assessing the model fit to the empirical data. The rule that functional relationships should occur in simple mathematical forms was also considered for determining the final form of the function. A statistical analysis was performed using the Statistica 13 PL statistical software package. The significance level in the analysis and inference was set at *p* = 0.05.

## Results and discussion

### The influence of head and deflector position and air velocity on the sowing value coefficient of variation

During the experiment, the seed weight distribution to each coulter was determined in the distributor head and the values of seed sowing quality coefficients were calculated, i.e. the coefficient of variation (CV) and coefficient of lateral seed sowing unevenness (*δ*) at three values of angles (0°, 5° and 10°) of distributor head tilt from the vertical and deflector tilt from the distributor head.

The experiment was conducted for three airflow velocities in a pneumatic seed drill system, i.e. 12.2 m/s, 14.4 m/s and 16.6 m/s. Values obtained from laboratory tests of the seed sowing unevenness index, as a function of the airflow velocity, distributor head and deflector inclination angles, were then statistically analyzed. Based on these findings, it was shown that the airflow velocity significantly influenced the unevenness of seed sowing, consistently with the findings obtained by Kumar et al.^40^, Gierz and Kęska^27^ and Yatskul et al.^1,30^.

Convergence can also be found between the results of the coefficient values of the seed sowing unevenness within the velocity increase range from 12.2 to 14.4 m/s with the results reported in the following articles^18,27,40^. The lowest mean coefficient value of seed sowing quality coefficients, i.e. coefficient of sowing variation (CV = 26.28%) and the coefficient of lateral seed sowing unevenness (*δ* = 24.42%) occurred for the airflow velocity of 14.4 m/s, the distributor head angle of 5° from the vertical and the deflector angle from the distributor head of 5°—a statistically significant difference at α = 0.05 (Tables 2, 3). The obtained results of the research clearly indicate that the seed sowing quality deterioration (lateral uniformity of seed sowing) is effectively prevented by means of a deflector plate with an adjustable tilt angle when the distributor head is inclined from the vertical up to 10°—calculated values coefficient of sowing variation (CV) and the coefficient of lateral seed sowing unevenness (*δ*) are close to the calculated values of these quality coefficients when the distribution head is vertical. An analysis of results also shows that an increase in air velocity in the pneumatic seed transport system to 16.6 m/s results in a deterioration of seed sowing quality, regardless of the applied combination of distribution head and deflector tilt angles.

**Table 2:** The average CV vs the deflector and distributor head tilt angle.

Theoretical sowing speed* v* (m/s)	Average CV (%)	Deflector tilt angle (°)	Distributor head tilt angle (°)	Average CV (%)
12.2	30.37 ± 5.95^C^	0	0	28.24 ± 0.98^b^
		0	5	35.38 ± 3.47^c^
		0	10	39.06 ± 1.31^c^
		5	5	21.40 ± 1.28^a^
		10	5	29.60 ± 0.60^b^
		10	10	28.56 ± 0.15^b^
14.4	26.28 ± 4.53^A^	0	0	25.02 ± 1.21^b^
		0	5	27.04 ± 0.84^b^
		0	10	33.61 ± 0.98^c^
		5	5	18.94 ± 1.80^a^
		10	5	27.35 ± 1.19^b^
		10	10	25.73 ± 0.36^b^
16.6	27.74 ± 4.86^B^	0	0	25.77 ± 0.61^b^
		0	5	29.48 ± 1.26^d^
		0	10	34.94 ± 1.10^e^
		5	5	19.29 ± 1.20^a^
		10	5	28.61 ± 0.27^ cd^
		10	10	26.24 ± 0.46^bc^

The average values (n) ± standard deviation; identical superscripts (A, B, C) I (a, b, c, d) show insignificant differences between the average values in columns according to Tukey’s HSD test for the factors under study.

**Table 3:** The average δ versus the deflector and distributor head tilt angle.

Theoretical sowing speed* v* (m/s)	Average *δ* (%)	Deflector tilt angle (°)	Distributor head tilt angle (°)	Average *δ* (%)
12.2	28.82 ± 5.15^C^	0	0	26.39 ± 0.80^b^
		0	5	33.23 ± 3.37^cd^
		0	10	34.95 ± 0.71^d^
		5	5	20.08 ± 1.48^a^
		10	5	29.48 ± 0.56^bc^
		10	10	28.77 ± 0.19^b^
14.4	24.42 ± 4.13^A^	0	0	25.04 ± 1.19^c^
		0	5	25.53 ± 1.01^c^
		0	10	29.61 ± 0.62^d^
		5	5	17.62 ± 1.80^a^
		10	5	27.19 ± 1.00^cd^
		10	10	21.51 ± 0.49^b^
16.6	26.06 ± 4.31^B^	0	0	24.76 ± 0.44^b^
		0	5	27.58 ± 1.19^c^
		0	10	30.84 ± 0.82^d^
		5	5	17.85 ± 1.29^a^
		10	5	28.31 ± 0.11^c^
		10	10	24.01 ± 0.61^b^

The average values (n) ± standard deviation; identical superscripts (A, B, C) I (a, b, c, d) show insignificant differences between the average values in columns according to Tukey’s HSD test for the factors under study.

The authors have observed that the angle of deflector tilt significantly affects the seed sowing evenness, which, according to the information reported in the literature^41,42^, can negatively affect the obtained crop yield. It is, therefore, reasonable to use a deflector to improve the quality of sowing with a pneumatic seed drill, especially when sowing in fields with an angle of terrain greater than 5° (Tables 2, 3).

Based on the obtained results, regression equations were derived with a posteriori elimination procedure and stepwise selection of insignificant variables and polynomial degree, describing the functional dependence of seed sowing quality, i.e. coefficient of sowing variation (CV)—Eq. 3 and the coefficient of lateral seed sowing unevenness (*δ*)—Eq. 4, depending on air stream velocity (*v*) in the pneumatic seed transport system, distribution head tilt from the vertical (*h*_*β*_) and the angle of the deflector in relation to the distributor head (*d*_*α*_).

3 $$ CV = - 16.38 \cdot v + 0.54 \cdot v^{2} - 4.38 \cdot d_{\alpha } + 0.39 \cdot d_{\alpha }^{2} + 0.95 \cdot h_{\beta } + 0.06 \cdot v \cdot d_{\alpha } \cdot h_{\beta } + 149.03 $$

4 $$ \delta = - 17.24 \cdot v + 0.57 \cdot v^{2} - 3.35 \cdot d_{\alpha } + 0.40 \cdot d_{\alpha }^{2} + 0.64 \cdot h_{\beta } - 0.14 \cdot h_{\beta } + 153.04 $$

Quadratic equations of seed sowing quality obtained from regression analysis are characterized by a very good model match with empirical data. The values of the coefficient of determination (R^2^) of the equations obtained was 0.94 and 0.91 respectively. These high values of the determination coefficient indicate a good match of the developed models with the empirical data, which allows prediction of the sowing process quality depending on the velocity of air stream (*v*) in the pneumatic seed transport system, distribution head tilt angle from the vertical (*h*_*β*_) and the deflector tilt angle relative to distribution head (*d*_*α*_).

**Figure 8:**
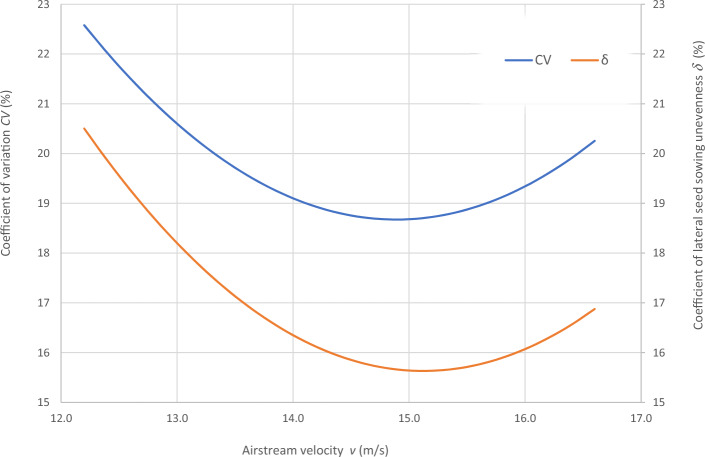
Seed sowing quality coefficients—coefficient of variation CV (%) and the coefficient of lateral seed sowing unevenness (δ) as a function of air velocity v (m/s) in the pneumatic seed transport system at constant values of the distributor head tilt angle from the vertical (hβ) 5° and the deflector tilt angle towards the distributor head (dα) 5°.

Graphical representation of quadratic equations describing the quality of seed sowing (coefficient of sowing variation (CV)—Eq. 3 and the coefficient of lateral seed sowing unevenness (*δ*)—Eq. 4), are shown in Fig. 8.

The experiment also determined the distribution of seed weight in the distribution head for individual coulters. The average seed weight for one coulter at airflow velocity of 12.2 m/s varied from 0.99 kg ± 0.012 kg to 1.12 kg ± 0.021 kg, depending on the distribution head and deflector angles. With an airflow velocity of 14.4 m/s, the mass of seed sown per coulter varied from 1.00 kg ± 0.018 kg to 1.19 kg ± 0.027 kg. In the third case, highest air flow velocity in the pneumatic system of 16.6 m/s, the average weight of seed directed to individual coulters varied from 1.00 kg ± 0.021 kg to 1.12 kg ± 0.032 kg (Table 4).

**Table 4:** The average seed weight for each coulter depending on the deflector setting angle (dα) and the setting angle of the seed-air stream distributor head (hβ).

Theoretical sowing speed *v* (m/s)	Deflector tilt angle *d*_*α*_ (°)	Head angle *h*_*β*_ (°)	Average seed weight per coulter (kg)
12.2	0	0	1.07 ± 0.0163
	0	5	1.06 ± 0.0136
	0	10	1.12 ± 0.0207
	5	5	1.04 ± 0.0363
	10	5	1.00 ± 0.0094
	10	10	0.99 ± 0.0119
14.4	0	0	1.00 ± 0.0178
	0	5	1.06 ± 0.0261
	0	10	1.13 ± 0.0291
	5	5	1.05 ± 0.0321
	10	5	1.01 ± 0.0045
	10	10	1.19 ± 0.0267
16.6	0	0	1.04 ± 0.0078
	0	5	1.06 ± 0.0235
	0	10	1.12 ± 0.0324
	5	5	1.05 ± 0.0351
	10	5	1.00 ± 0.0208
	10	10	1.11 ± 0.0252

± Standard deviation.

**Figure 9:**
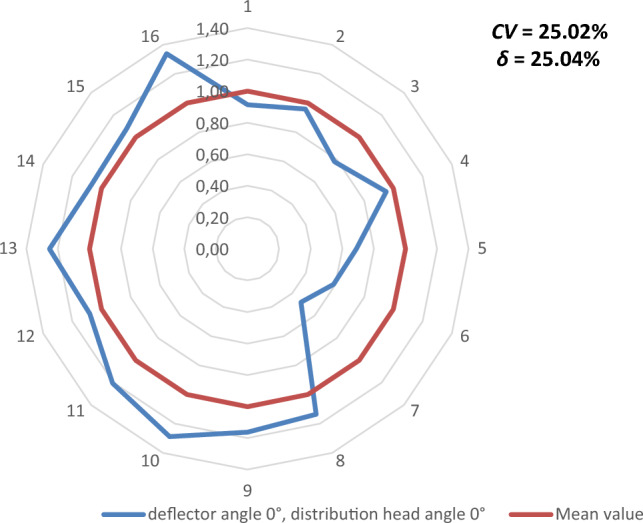
The seed distribution at a deflector angle of 0°, distribution head angle of 0° and air stream velocity of 14.4 m/s.

**Figure 10:**
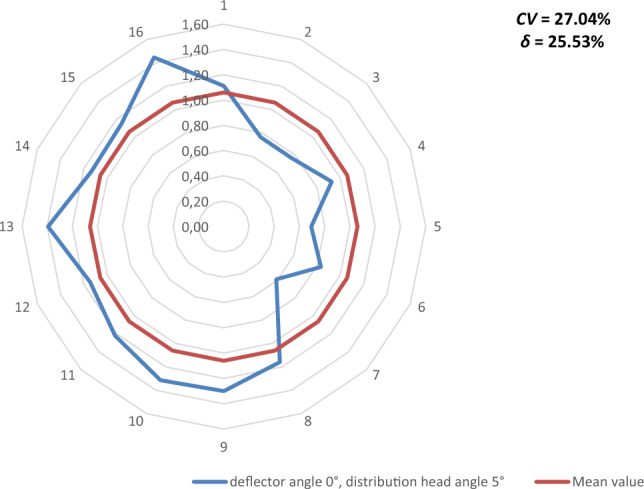
The seed distribution at a deflector angle of 0°, distribution head angle of 5° and air stream velocity of 14.4 m/s.

**Figure 11:**
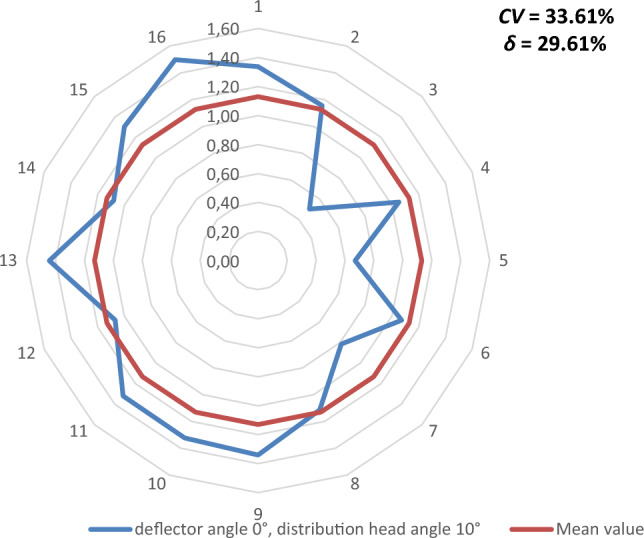
The seed distribution at a deflector angle of 0°, distribution head angle of 10° and air stream velocity of 14.4 m/s.

**Figure 12:**
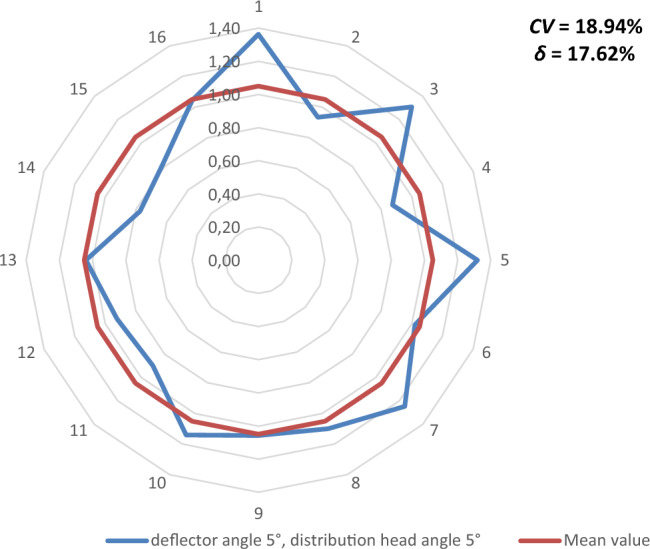
The seed distribution at a deflector angle of 5°, distribution head angle of 5° and air stream velocity of 14.4 m/s.

**Figure 13:**
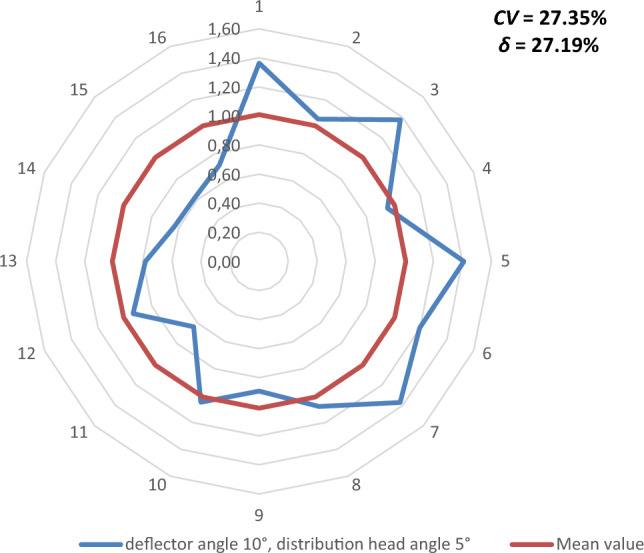
The seed distribution at a deflector angle of 5°, distribution head angle of 5° and air stream velocity of 14.4 m/s.

**Figure 14:**
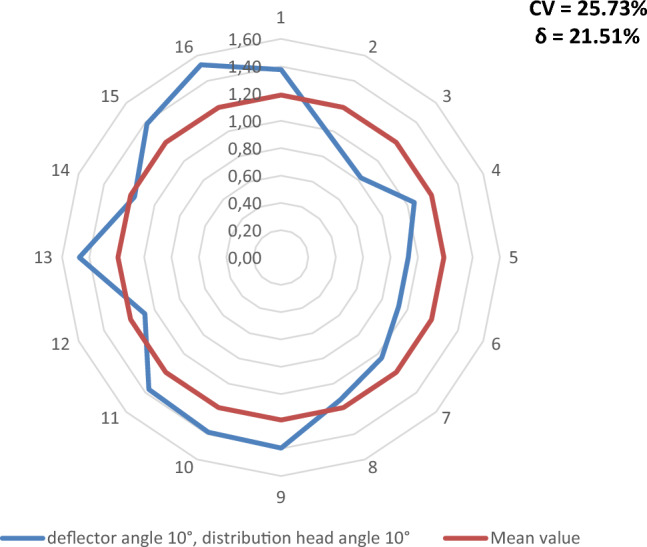
The seed distribution at a deflector angle of 10°, distribution head angle of 10° and air stream velocity of 14.4 m/s.

Figures 9, 10, 11, 12, 13 and 14 show the seed weight distribution for each of the 16 coulters as a function of deflector tilt angle (*d*_*α*_) and tilt angle of the seed-air stream distributor head (*h*_*β*_) along with the calculated values of the seed quality coefficients, i.e. coefficient of sowing variation (CV) and the coefficient of lateral seed sowing unevenness (*δ*) and air stream velocity 14.4 m/s—at which the highest seed sowing evenness (the lowest coefficient values CV and* δ*).

The highest CV and *δ* (33.61% and 29.61% respectively), i.e. the most uneven seed sowing was observed with a distribution head angle of 10° from the vertical and a deflector angle of 0° (this value of deflector angle represents the classic distribution head configuration—without deflector) (Fig. 11). Deflector angle adjustment (*d*_*α*_) from 0° to 10°, with a distribution head angle of 10° from the vertical (Fig. 14) improved the seed sowing quality—coefficient values CV and* δ* decreased to 25.73% and 21.51% respectively. The obtained CV and *δ* coefficient values clearly show the effectiveness of the proposed solution related to the use of a deflector in the distribution head. The relevance of the proposed solution is also confirmed by the CV and* δ* values (18.94 and 21.51 respectively) obtained at a deflector angle of 5° and a distribution head angle of 5° from the vertical (Fig. 12). The CV and *δ* values when a deflector were used, compared to the variant without a deflector and the same head seed-air stream divider angle setting value of 5° (*h*_*β*_) were 25.73% and 21.51%, respectively.

## Conclusions

- The developed and manufactured innovative distributor head with a deflector that tilts in two planes, designed to improve the distribution evenness of the air stream transporting seed to individual coulters in pneumatic seed drills, provided positive results in this experiment.
- Changing the deflector tilt angle significantly affects the value of the coefficient of lateral seed sowing unevenness and can compensate for the effect of the seed drill tilt and the supply conduit spreader curvature. Change in the distributor head angle of tilt from the vertical position (0°) by 5° and 10°, accompanied with appropriate change in the deflector angle by the above given angles, that is, 5° and 10° caused reduction in the value of the sowing lateral unevenness indicator from about 20% up to above 40%, depending on the working speed and indicator applied.
- The research result analysis shows that the air stream velocity transporting the seed has a significant influence on the uniformity of air stream distribution in the distributor head. The lowest value of the seed sowing quality coefficient, i.e. coefficient of sowing variation (CV) and the coefficient of lateral seed sowing unevenness (*δ*) was obtained for air velocity of 14.4 m/s. Changing the air velocity from 14.4 to 12.2 m/s or 16.6 m/s significantly worsened the seed distribution evenness in the distributor head—increase in the value of indicators of seed sowing lateral unevenness within the range from about 5% to about 18%.
- The second-degree equations of seed sowing quality determined from a multiple regression analysis, i.e. coefficient of sowing variation (CV) and the coefficient of lateral seed sowing unevenness (*δ*), is characterized by a high percentage of explained variability (R^2^ 0.94 and 0.91, respectively). This definitely confirms usefulness of the equations for prediction of the seed sowing process quality as a function of the airflow stream velocity (*v*) in the pneumatic seed transport system, the deviation of the distributor head from the vertical (*h*_*β*_) and the deflector tilt angle relative to the distributor head (*d*_*α*_).
- The research results indicate that it is reasonable to improve the quality of sowing with pneumatic seed drills, especially when sowing in areas with an over 5° inclination. This requires continuous measurement of the asymmetry coefficient of the seed stream distribution in the head seed distributor and the use of a system for automatic adjustment of the deflector angle that can be performed by the increasingly used 3D printing method^43^ or by incremental manufacturing^44^. These topics may be the subject of further research.

## Data Availability

Correspondence and requests for materials should be addressed to Łukasz Gierz (Ł.G) lukasz.gierz@put.poznan.pl.
